# Personality traits and biological aging: the mediating role of coping behavior

**DOI:** 10.3389/fpsyg.2026.1876764

**Published:** 2026-10-07

**Authors:** Shirui Peng, Zixuan Li, Tao Huang, Hailun Liang

**Affiliations:** 1 School of Public Administration and Policy, Renmin University of China, Beijing, China; 2 Beijing Center for Disease Prevention and Control, Beijing, China; 3 Department of Epidemiology & Biostatistics, School of Public Health, Peking University, Beijing, China; 4 School of Population and Health, Renmin University of China, Beijing, China

**Keywords:** Biological aging, Coping behavior, Genetic risk, Personality traits, PhenoAge acceleration

## Abstract

**Background:**

Evidence linking Big Five personality traits to clinical biomarker-based PhenoAge acceleration remains limited, and the behavioral pathways underlying these associations are not well understood.

**Objective:**

This study examined the associations between Big Five personality traits and PhenoAge acceleration (PhenoAA), tested health-related coping behavior as a statistical mediator, and explored differences across genetic risk strata.

**Methods:**

We conducted a cross-sectional analysis of 119,250 European-ancestry participants from the UK Biobank. PhenoAA was derived from clinical biomarker-based PhenoAge after adjusting for chronological age. Multivariable linear regression, mediation analysis, inverse-probability-weighted sensitivity analysis, and exploratory analyses stratified by a genetic risk score (GRS) based on 29 single-nucleotide polymorphisms were performed.

**Results:**

Neuroticism was positively associated with PhenoAA (*β* = 0.038, *p* < 0.001). Extraversion (*β* = −0.301, *p* < 0.001), conscientiousness (*β* = −0.314, *p* < 0.001), and agreeableness (*β* = −0.037, *p* < 0.01) were inversely associated with PhenoAA, while openness was not significant (*β* = −0.023, *p* > 0.05). Coping behavior statistically mediated the associations for neuroticism, extraversion, conscientiousness, and agreeableness, accounting for 15.4, 19.5, 12.3, and 28.2% of the total associations, respectively. Weighted sensitivity analyses showed similar patterns. In GRS-stratified analyses, neuroticism, extraversion, and conscientiousness showed broadly consistent associations across low- and high-GRS groups. Agreeableness was inversely associated with PhenoAA only in the high-GRS group. Interaction tests did not provide evidence of genetic effect modification.

**Conclusion:**

Big Five personality traits were associated with PhenoAA, and coping behavior partly accounted for several of these associations. These findings support a biopsychosocial view of biological aging, but they should be interpreted as associations rather than causal relationships.

## Introduction

Aging is a dynamic and multifactorial process marked by gradual decline across multiple physiological systems. Chronological age is widely used, but it does not fully capture individual differences in the pace of biological aging. These differences are important because biological aging is closely related to healthspan, morbidity, and mortality (López-Otín et al., 2013; Kennedy et al., 2014). Clinical biomarker-based PhenoAge measures biological aging. It was developed from chronological age and routinely assessed clinical biomarkers and has been applied in large population studies, including the National Health and Nutrition Examination Survey (NHANES) and UK Biobank analyses (Levine et al., 2018; Liu et al., 2018; Gao et al., 2023). PhenoAge combines chronological age with biomarkers that reflect metabolic, inflammatory, immune, and organ-function status. PhenoAge acceleration (PhenoAA) extends this measure by indicating whether an individual’s PhenoAge is higher or lower than expected for their chronological age, with higher values indicating accelerated biological aging. PhenoAge and PhenoAA have been linked to morbidity, disease burden, and mortality risk in population-based studies (Levine et al., 2018; Liu et al., 2018; Belsky et al., 2020; Gao et al., 2023). PhenoAA has also been associated with genetic, environmental, and psychosocial factors (Lu et al., 2019; Kuo et al., 2021; Stephan et al., 2021; Gao et al., 2023). These features make PhenoAA a useful outcome for examining how psychological and behavioral factors relate to biological aging.

Personality traits are important psychosocial factors that may influence biological aging. The Five-Factor Model describes five broad personality domains: neuroticism, extraversion, openness, agreeableness, and conscientiousness (Costa and McCrae, 1992). These traits shape how individuals appraise stress, regulate behavior, and respond to their social and physical environments. They are also closely related to long-term health outcomes (Friedman and Kern, 2014; Mroczek and Spiro, 2007; Roberts et al., 2007; Hampson and Friedman, 2008). For example, higher neuroticism scores have been associated with cardiovascular disease, systemic inflammation, and premature mortality (Jokela et al., 2013; Sutin et al., 2013). In contrast, conscientiousness has been linked to health-promoting behaviors, better physiological regulation, and greater longevity (Kern and Friedman, 2008; Turiano et al., 2012). These findings suggest that personality traits may be reflected in biological aging through repeated stress exposure, behavioral regulation, and physiological stress responses. However, evidence linking Big Five traits to clinical biomarker-based PhenoAge acceleration remains limited. The behavioral processes that may connect personality traits with PhenoAA are also not fully understood.

Coping behavior offers a plausible behavioral pathway to link personality traits with PhenoAA. Coping refers to the cognitive and behavioral efforts individuals use to manage stressful demands (Lazarus and Folkman, 1984; Taylor and Stanton, 2007). Personality traits may shape these efforts because individuals differ in how they appraise stress and respond to it. In this sense, coping behavior may be one way through which stable personality traits are expressed in daily life. For example, higher neuroticism has been linked to rumination, avoidance, and emotion-focused coping, whereas conscientiousness has been associated with more planned and problem-focused responses (Bolger and Zuckerman, 1995; Connor-Smith and Flachsbart, 2007; Vollrath, 2001; Carver and Connor-Smith, 2010). Population-based studies further suggest that coping behavior can include observable stress-related behaviors in addition to cognitive and emotional strategies (Mezuk et al., 2017; Bergmans et al., 2021). These behaviors may overlap with health behaviors, but they are treated as coping behavior when they are studied as responses to stress or emotional distress. This distinction is important for research on biological aging, as repeated coping responses may contribute to metabolic, inflammatory, and physiological regulation over time. Previous studies have linked coping behavior with depressive symptoms, inflammation, and metabolic risk (Charles et al., 2013; Leger et al., 2016; Sutin et al., 2016). Some evidence also suggests that stress-related behaviors may help explain associations between personality and aging biomarkers, such as telomere length (O'Donovan et al., 2009). However, whether coping behavior statistically accounts for the association between Big Five personality traits and PhenoAA remains unclear.

Although coping behavior may serve as a behavioral pathway linking personality traits with PhenoAA, biological aging is unlikely to be shaped by behavioral factors alone. Genetic susceptibility also contributes to individual differences in biological aging. Previous studies have shown that genetic factors are associated with biological age acceleration, including PhenoAge acceleration (Lu et al., 2019; Kuo et al., 2021; Belsky et al., 2020). These genetic signals may reflect biological processes related to inflammation, immune function, and metabolic regulation. Broader work in social genomics also suggests that psychological, behavioral, and genetic factors should be considered together when studying health and aging (Cole, 2014). In the present study, the PhenoAge acceleration genetic risk score (GRS) was used to represent inherited susceptibility to accelerated PhenoAge. GRS-stratified analyses provided an exploratory approach to examine whether the associations between personality traits and PhenoAA were similar or different across low and high genetic risk backgrounds.

Taken together, this study addressed three related questions. First, we examined whether Big Five personality traits were associated with PhenoAge acceleration (PhenoAA) in a large population-based sample from the UK Biobank. Second, we tested the mediating role of coping behavior in these associations. Third, we conducted exploratory analyses stratified by the PhenoAge acceleration GRS to examine whether the associations between personality traits and PhenoAA differed across low and high genetic risk groups. This study extends previous research by moving beyond direct associations between personality traits and biological aging. It examines coping behavior as a potential behavioral pathway. It also considers whether these associations appear consistent across different levels of inherited susceptibility. This approach may help clarify how psychological traits, coping behavior, and genetic risk are related to biological aging.

## Methods

### Research design and participants

The present study employed an observational cross-sectional design and analyzed data from the UK Biobank. The primary objective was to examine the associations between Big Five personality traits and PhenoAge acceleration and to assess whether health-related coping behavior statistically mediated these associations.

The UK Biobank recruited a total of 502,411 participants. Of these, 15,002 participants were excluded because genotype data required to construct the PhenoAge acceleration genetic risk score (GRS) were unavailable, leaving 487,409 participants with available GRS-related genotype data. We then excluded 14,797 participants of non-European ancestry, resulting in 472,612 European-ancestry participants. After excluding 47 participants with missing PhenoAge acceleration phenotype data, 472,565 participants were eligible for complete-case screening. We further excluded 353,315 participants with missing Big Five personality trait measures, coping behavior scores, or covariates. The final complete-case structural equation modeling (SEM) analytic sample included 119,250 participants. Among participants eligible for complete-case screening, exclusions were primarily due to missing personality trait measures or coping behavior scores.

All participants provided written informed consent. The UK Biobank study received approval from the North West Multi-centre Research Ethics Committee as a Research Tissue Bank (Ref: 11/NW/0382) (see Figure 1).

**Figure 1:**
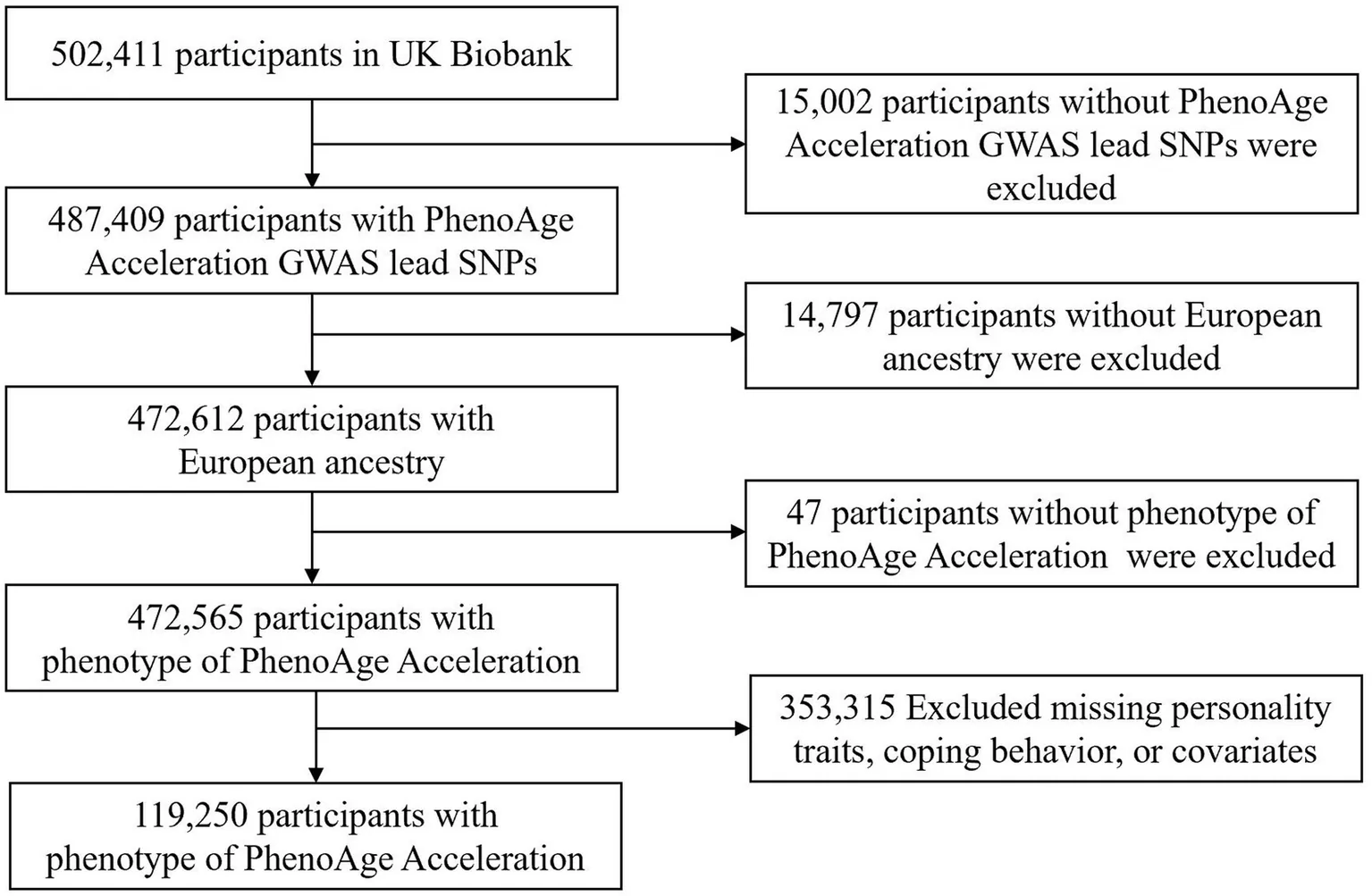
Participant selection. Numbers represent sequential exclusions at each step.

### Measurement tools

#### PhenoAge and PhenoAge acceleration

PhenoAge was used as a clinical biomarker-based measure of biological aging. Following the methodologies established by Levine et al. (2018), Liu et al. (2018), and the UK Biobank data-cleaning and variable-derivation procedures used by Gao et al. (2023), PhenoAge was calculated using chronological age and nine routinely assessed clinical biomarkers: albumin, alkaline phosphatase, creatinine, C-reactive protein, glucose, mean cell volume, red cell distribution width, white blood cell count, and lymphocyte proportion. These biomarkers were originally selected from a broader panel of clinical indicators identified through penalized regression models for mortality prediction, and they have been widely used to capture multisystem physiological aging.

PhenoAge acceleration (PhenoAA) is a measure used to quantify whether an individual’s PhenoAge was higher or lower than expected for their chronological age. Consistent with previous studies on biological age acceleration in the UK Biobank, PhenoAA was derived as the residual from a linear regression model in which PhenoAge was regressed on chronological age. Positive PhenoAA values indicate that an individual’s PhenoAge was higher than expected for their chronological age, whereas negative values indicate lower-than-expected PhenoAge. As PhenoAA was based on regression residuals, its mean is expected to be close to zero in the fitted sample. We used PhenoAA as the primary outcome variable in this study.

The present study used clinical biomarker-based PhenoAge rather than DNA methylation-derived PhenoAge. Therefore, standard adjustments for methylation-derived blood cell-type composition were not directly applicable. However, white blood cell count and lymphocyte proportion are included as components of the PhenoAge algorithm.

#### Genetic risk score for PhenoAge acceleration

The genetic risk score (GRS) for PhenoAge acceleration was used to represent individual genetic susceptibility to accelerated PhenoAge. The score was constructed from 29 single-nucleotide polymorphisms (SNPs) previously associated with PhenoAge acceleration in genome-wide association studies (Kuo et al., 2021). Genotyping, imputation, and quality control procedures in the UK Biobank have been described previously (Sudlow et al., 2015).

For each participant, every SNP was coded as 0, 1, or 2 according to the number of risk alleles. Each allele count was weighted by the corresponding effect size reported in the previous genome-wide association study, and weighted values were summed to generate the GRS. Higher GRS values indicate greater genetic susceptibility to accelerated PhenoAge. In this study, the GRS was treated as a continuous measure of genetic risk.

#### Personality traits

Personality traits were operationalized according to the Five-Factor Model, which includes neuroticism, extraversion, conscientiousness, agreeableness, and openness to experience (John and Srivastava, 1999; McCrae and Costa, 1999). Since the UK Biobank did not administer a full Big Five inventory, we constructed trait-level proxy measures from available baseline self-report items. This coding strategy followed the UK Biobank personality proxy approach proposed by Ruijter et al. (2022), who mapped UK Biobank questionnaire items onto personality-related constructs, particularly diligence and sociability. Similar proxy-based approaches have also been used in large-scale population studies when conventional psychometric inventories are unavailable (Elovainio et al., 2017; Hakulinen et al., 2015).

Neuroticism was assessed using the validated 12-item Eysenck Personality Questionnaire-Revised Short Form, as described by Smith et al. (2013). Items were coded such that higher scores indicated greater levels of neuroticism and were summed to generate a continuous neuroticism score.

For the other four traits, proxy indicators were constructed based on prior mappings between UK Biobank items and Big Five-related domains, as outlined by Ruijter et al. (2022). The proxy indicators were selected to correspond to the four non-neuroticism Big Five traits. Diligence, persistence, and risk avoidance were used to approximate conscientiousness. Sociability and social engagement were used to approximate extraversion. Interpersonal warmth and trust were used to approximate agreeableness. Curiosity and receptivity to new experiences were used to approximate openness. Higher scores indicated higher levels of the corresponding trait. All personality traits were modeled as continuous variables.

#### Coping behavior

Coping behavior was defined as behavioral responses that individuals may use when dealing with stressful experiences. This definition follows classic coping theory (Lazarus and Folkman, 1984) and prior population-based studies that measured coping through observable stress-related behaviors (Mezuk et al., 2017; Bergmans et al., 2021; Ghosn et al., 2020). In this study, these indicators were used to represent observable stress-related coping behaviors, in line with prior population-based studies that conceptualized alcohol use, medication use, overeating, smoking, physical activity, and help-seeking as behavioral responses to stress or emotional distress.

The coping behavior score was developed using six UK Biobank indicators that corresponded to domains used in previous studies of stress-coping behavior. These domains included alcohol consumption, drug or medication use, overeating, smoking, physical activity, and professional help-seeking.

The six components were coded as follows: (1) low-risk alcohol consumption, defined as a weekly intake of less than 14 g of pure alcohol for women and less than 28 g for men; (2) no drug or medication use; (3) no overeating; (4) non-current smoking status, defined as never smoking or being a former smoker; (5) sufficient physical activity, defined as engaging in at least 150 min of moderate-intensity activity, 75 min of vigorous-intensity activity, or an equivalent combination per week; and (6) seeking professional help for mental distress, psychological problems, or unusual experiences. A regular pattern of moderate-intensity activity at least 5 days per week or vigorous-intensity activity at least once a week was also coded as sufficient physical activity.

Each component was coded as 1 if the participant met the prespecified criteria and 0 otherwise. The six binary indicators were summed to create a coping behavior score that ranges from 0 to 6. A higher score indicates that a participant met a greater number of the prespecified coping behavior criteria. The total score represents the number of prespecified coping behavior criteria met by each participant, while allowing the individual components to retain distinct psychological and biological meanings.

The score was calculated as an unweighted sum to maintain a transparent and reproducible scoring approach, consistent with prior epidemiological studies using binary or count indicators of coping behavior. The score was used as a composite profile of coping-related behaviors.

#### Statistical analysis

All analyses were conducted using Stata version 17. Participant characteristics were summarized as means and standard deviations for continuous variables and frequencies and percentages for categorical variables. Sex differences in baseline characteristics were assessed using independent samples *t*-tests for continuous variables and chi-square tests for categorical variables, with two-sided *p*-values reported where applicable.

Multivariable linear regression models were used to examine the associations between each Big Five personality trait and PhenoAge acceleration (PhenoAA). Separate models were fitted for neuroticism, extraversion, conscientiousness, agreeableness, and openness. All models were adjusted for chronological age, sex, Townsend deprivation index, coronary heart disease, hypertension, and diabetes. A Bonferroni-corrected threshold of *p* < 0.01 was used for the primary association analyses of the five personality traits. Nominal two-sided *p*-values were also reported.

We then conducted cross-sectional statistical mediation analyses to examine whether coping behavior statistically accounted for part of the associations between personality traits and PhenoAA. Mediation models were fitted separately for each personality trait, with the personality trait specified as the exposure, coping behavior as the mediator, and PhenoAA as the outcome. The same covariates were included in all mediation models. Total, direct, and indirect associations were estimated, and the proportion mediated was calculated as the indirect association divided by the total association. These analyses were used to describe the statistical decomposition of cross-sectional associations.

To evaluate potential missing data-related selection, we compared complete-case participants with those excluded because of missing data. Inverse probability weighting was then used as a sensitivity analysis. The probability of inclusion in the complete-case analytic sample was estimated using available baseline characteristics, and stabilized inverse probability weights were applied to the mediation models. Weighted results were compared with complete-case results to assess whether the direction and magnitude of the main mediation estimates changed materially.

Finally, exploratory stratified analyses were conducted based on PhenoAge acceleration genetic risk. Participants were classified into low- and high-GRS groups using the median value of the PhenoAge acceleration GRS in the complete-case analytic sample. Median-based stratification has been used in previous genetic risk score and polygenic risk score studies to compare groups with relatively lower and higher genetic susceptibility (Nurnberger Jr et al., 2021; French et al., 2015; Restrepo-Lozano et al., 2022; Watt et al., 2019; Jaramillo-Ospina et al., 2022). This approach also provided two comparably sized strata for the present exploratory analyses. The median GRS in the complete-case sample was 21. Participants with GRS values below 21 were classified as the low-GRS group, and those with GRS values of 21 or higher were classified as the high-GRS group.

Within each GRS stratum, multivariable linear regression models were used to estimate the associations between each Big Five personality trait and PhenoAA. These models were adjusted for chronological age, sex, Townsend deprivation index, coronary heart disease, hypertension, and diabetes. Interaction terms between each personality trait and GRS group were then tested in the complete-case analytic sample to assess the effect modification.

## Results

### Characteristics of participants

Table 1 shows the basic characteristics of the 119,250 participants. The mean PhenoAge was 44.20 ± 9.39 years, and the mean PhenoAge acceleration was approximately zero (−0.00 ± 5.03), as expected for a residual-based measure. The mean GRS of PhenoAge acceleration was 20.73 ± 2.43. PhenoAge was higher among male participants (45.56 ± 9.62 years) than among female participants (43.10 ± 9.05 years; *p* < 0.001). PhenoAge acceleration was also higher among male participants (0.68 ± 4.86) than among female participants (−0.55 ± 5.10; *p* < 0.001). In contrast, the GRS of PhenoAge acceleration did not differ significantly by sex. The mean GRS was 20.73 ± 2.43 among male participants and 20.74 ± 2.44 among female participants (*p* = 0.658).

**Table 1:** Characteristics of study participants.

Characteristics	Total (*n* = 119,250)	Male (*n* = 53,242)	Female (*n* = 66,008)	*p*
Townsend deprivation index	−1.76 (2.80)	−1.83 (2.80)	−1.71 (2.80)	<0.001
Major diseases				
Coronary heart disease				
No	115,420 (96.79%)	50,402 (94.67%)	65,018 (98.50%)	<0.001
Yes	3,830 (3.21%)	2,840 (5.33%)	990 (1.50%)	
Hypertension				<0.001
No	91,536 (76.76%)	38,226 (71.80%)	53,310 (80.76%)	
Yes	27,714 (23.24%)	15,016 (28.20%)	12,698 (19.24%)	
Diabetes				<0.001
No	115,216 (96.62%)	50,775 (95.37%)	64,441 (97.63%)	
Yes	4,034 (3.38%)	2,467 (4.63%)	1,567 (2.37%)	
Coping behavior				<0.001
0	38 (0.03%)	17 (0.03%)	21 (0.03%)	
1	532 (0.45%)	251 (0.47%)	281 (0.43%)	
2	3,767 (3.16%)	1,688 (3.17%)	2,079 (3.15%)	
3	17,380 (14.57%)	7,366 (13.83%)	10,014 (15.17%)	
4	42,682 (35.79%)	18,802 (35.31%)	23,880 (36.18%)	
5	43,146 (36.18%)	20,316 (38.16%)	22,830 (34.59%)	
6	11,705 (9.82%)	4,802 (9.02%)	6,903 (10.46%)	
PhenoAge	44.20 (9.39)	45.56 (9.62)	43.10 (9.05)	<0.001
PhenoAge acceleration	−0.00 (5.03)	0.68 (4.86)	−0.55 (5.10)	<0.001
GRS of PhenoAge acceleration	20.73 (2.43)	20.73 (2.43)	20.74 (2.44)	0.658

The coping behavior score ranges from 0 to 6 and represents the number of prespecified coping behavior criteria met by each participant. The six criteria were low-risk alcohol consumption, no drug or medication use, no overeating, non-current smoking, sufficient physical activity, and seeking professional help for mental distress, psychological problems, or unusual experiences. Each criterion was coded as 1 if met and 0 otherwise, as described in the Measurement tools section.

A comparison between complete-case participants and excluded participants is presented in Supplementary Table S1. Given the observed differences between the two groups, inverse probability weighting was used in sensitivity analyses to evaluate the potential influence of missing-data-related selection.

Continuous variables are presented as mean (standard deviation), and categorical variables are presented as *n* (%). *p*-values compare male and female participants. Independent-samples *t*-tests were used for continuous variables, and Pearson’s chi-square tests were used for categorical variables. All *p*-values are two-sided. GRS, genetic risk score.

### Total associations between personality traits and PhenoAge acceleration

We conducted multivariable linear regression models to examine the total associations between personality traits and PhenoAge acceleration, as shown in Table 2. After adjusting for age, sex, Townsend deprivation index, coronary heart disease, hypertension, and diabetes, neuroticism was positively associated with PhenoAge acceleration (*β* = 0.038, *p* < 0.001). In contrast, extraversion (*β* = −0.301, *p* < 0.001), conscientiousness (*β* = −0.314, *p* < 0.001), and agreeableness (*β* = −0.037, *p* < 0.01) were inversely associated with PhenoAge acceleration. Openness was not significantly associated with PhenoAge acceleration (*β* = −0.023, *p* > 0.05).

**Table 2:** Associations between personality traits and PhenoAge acceleration.^a^

Variable	PhenoAA Model 1	PhenoAA Model 2	PhenoAA Model 3	PhenoAA Model 4	PhenoAA Model 5
Neuroticism	0.038*** (3.30)				
Extraversion		−0.301*** (−18.51)			
Conscientiousness			−0.314*** (−19.21)		
Agreeableness				−0.037** (−3.27)	
Openness					−0.023 (−1.14)
Age	−0.021*** (−11.03)	−0.017*** (−8.93)	−0.018*** (−9.13)	−0.021*** (−11.13)	−0.022*** (−11.42)
Sex	1.078*** (37.63)	1.034*** (36.12)	1.002*** (34.83)	1.071*** (37.49)	1.074*** (37.36)
TDI	0.096*** (17.94)	0.093*** (17.40)	0.089*** (16.59)	0.096*** (17.92)	0.097*** (18.04)
Coronary heart disease	0.539*** (5.70)	0.520*** (5.52)	0.510*** (5.42)	0.539*** (5.71)	0.545*** (5.77)
Hypertension	1.130*** (30.45)	1.115*** (30.19)	1.123*** (30.40)	1.130*** (30.47)	1.136*** (30.66)
Diabetes	3.006*** (28.72)	2.963*** (28.36)	2.965*** (28.36)	3.007*** (28.73)	3.007*** (28.73)
Constant	0.444*** (4.01)	0.974*** (8.82)	1.160*** (10.28)	0.644*** (5.70)	0.576*** (5.10)

^a^Models adjusted for age, sex, townsend deprivation index, coronary heart disease, hypertension, and diabetes. PhenoAA, PhenoAge acceleration; TDI, Townsend deprivation index.

^*^p < 0.05, ^**^p < 0.01, ^***^p < 0.001 (two-tailed tests).

### The mediating role of coping behavior

We further examined whether health-related coping behavior statistically accounted for the associations between personality traits and PhenoAge acceleration. Higher coping behavior scores indicated that participants met a greater number of the prespecified coping behavior criteria. As shown in Supplementary Table S2, higher neuroticism scores were associated with lower coping behavior scores. In contrast, higher extraversion, conscientiousness, and agreeableness scores were associated with higher coping behavior scores. Openness was not clearly associated with coping behavior. Higher coping behavior scores were also associated with lower PhenoAge acceleration after adjustment for personality traits and covariates.

As shown in Table 3, Supplementary Table S2, the indirect association through coping behavior was significant for neuroticism, extraversion, conscientiousness, and agreeableness. For neuroticism, the indirect association was positive (*β* = 0.006, *p* < 0.001), accounting for 15.4% of the total association. In contrast, the indirect associations were negative for extraversion (*β* = −0.059, *p* < 0.001), conscientiousness (*β* = −0.039, *p* < 0.001), and agreeableness (*β* = −0.010, *p* < 0.001), accounting for 19.5, 12.3, and 28.2% of their total associations, respectively. Openness showed no evidence of statistical mediation through coping behavior.

**Table 3:** Cross-sectional statistical mediation by coping behavior.

Personality trait	Total	Direct	Indirect	Proportion
Neuroticism	0.038***	0.032**	0.006***	15.4%**
Extraversion	−0.301***	−0.243***	−0.059***	19.5%***
Conscientiousness	−0.314***	−0.275***	−0.039***	12.3%***
Agreeableness	−0.037**	−0.026*	−0.010***	28.2%**
Openness	−0.023	−0.024	0.002	

The associations of personality traits with PhenoAge acceleration and the mediating effect of coping behavior. ^*^p < 0.05, ^**^p < 0.01, and ^***^p < 0.001.

### Sensitivity analysis for missing-data-related selection

Because the primary analyses were based on a complete-case sample, we compared included participants with those excluded because of missing data. As shown in Supplementary Table S1, complete-case participants differed from excluded participants in several baseline characteristics, including age, Townsend deprivation index, PhenoAge acceleration, and major cardiometabolic conditions. These differences suggested the possibility of missing-data-related selection.

We therefore conducted inverse probability weighted mediation analyses as a sensitivity analysis. As shown in Supplementary Table S3, the inverse-probability-weighted results were broadly consistent with the complete-case mediation analyses. The directions of the total, direct, indirect, a-path, and b-path associations remained unchanged for neuroticism, extraversion, conscientiousness, and agreeableness. Openness continued to show no clear evidence of statistical mediation. These findings suggest that the main mediation patterns were not fully explained by missing-data-related sample selection.

### Stratified associations by genetic risk of PhenoAge acceleration

Participants were stratified by the median value of the PhenoAge acceleration GRS. The low-GRS group included 53,988 participants with GRS values below the median, and the high-GRS group included 65,262 participants with GRS values at or above the median. As shown in Table 4, neuroticism was positively associated with PhenoAge acceleration in both the low- and high-GRS groups (*β* = 0.037, *t* = 2.17, *p* < 0.05; *β* = 0.037, *t* = 2.46, *p* < 0.05). Extraversion was inversely associated with PhenoAge acceleration in both strata (*β* = −0.315, *t* = −12.88, *p* < 0.001; *β* = −0.290, *t* = −13.29, *p* < 0.001). Conscientiousness also showed inverse associations in both the low- and high-GRS groups (*β* = −0.311, *t* = −12.72, *p* < 0.001; *β* = −0.316, *t* = −14.40, *p* < 0.001).

**Table 4:** Associations between personality traits and PhenoAge acceleration stratified by genetic risk of PhenoAge acceleration.^a^

Variable	Low GRS	High GRS	Low GRS	High GRS	Low GRS	High GRS	Low GRS	High GRS	Low GRS	High GRS
Neuroticism	0.037* (2.17)	0.037* (2.46)								
Extraversion			−0.315*** (−12.88)	−0.290*** (−13.29)						
Conscientiousness					−0.311*** (−12.72)	−0.316*** (−14.40)				
Agreeableness							−0.030 (−1.79)	−0.041** (−2.77)		
Openness									−0.029 (−0.97)	−0.017 (−0.66)
Age	−0.023*** (−8.02)	−0.020*** (−7.66)	−0.019*** (−6.50)	−0.016*** (−6.20)	−0.019*** (−6.79)	−0.016*** (−6.21)	−0.023*** (−8.12)	−0.020*** (−7.70)	−0.024*** (−8.29)	−0.021*** (−7.94)
Sex	1.079*** (25.15)	1.077*** (27.98)	1.034*** (24.16)	1.034*** (26.84)	1.004*** (23.34)	0.999*** (25.84)	1.072*** (25.07)	1.070*** (27.87)	1.075*** (25.00)	1.072*** (27.76)
TDI	0.095*** (11.73)	0.097*** (13.61)	0.092*** (11.37)	0.094*** (13.21)	0.088*** (10.85)	0.090*** (12.59)	0.095*** (11.73)	0.097*** (13.58)	0.096*** (11.79)	0.097*** (13.69)
Coronary heart disease	0.883*** (5.93)	0.253* (2.09)	0.873*** (5.89)	0.226 (1.87)	0.852*** (5.74)	0.225 (1.87)	0.884*** (5.94)	0.252* (2.09)	0.890*** (5.98)	0.258* (2.13)
Hypertension	1.137*** (20.62)	1.129*** (22.50)	1.118*** (20.37)	1.117*** (22.36)	1.131*** (20.60)	1.121*** (22.45)	1.138*** (20.65)	1.128*** (22.49)	1.142*** (20.76)	1.134*** (22.65)
Diabetes	3.075*** (19.46)	2.944*** (21.14)	3.031*** (19.21)	2.901*** (20.87)	3.026*** (19.16)	2.911*** (20.93)	3.076*** (19.46)	2.945*** (21.14)	3.077*** (19.47)	2.945*** (21.14)
_cons	0.431** (2.61)	0.462** (3.08)	0.979*** (6.01)	0.975*** (6.49)	1.142*** (6.81)	1.180*** (7.74)	0.611*** (3.65)	0.675*** (4.40)	0.574*** (3.42)	0.583*** (3.81)

^a^Models adjusted for age, sex, townsend deprivation index, coronary heart disease, hypertension, and diabetes. GRS, genetic risk score; TDI, Townsend deprivation index.

*p < 0.05, ^**^p < 0.01, ^***^p < 0.001 (two-tailed tests).

For agreeableness, the association differed descriptively across GRS strata. Agreeableness was not significantly associated with PhenoAge acceleration in the low-GRS group, but it was inversely associated with PhenoAge acceleration in the high-GRS group (*β* = −0.030, *t* = −1.79, *p* > 0.05; *β* = −0.041, *t* = −2.77, *p* < 0.01). Openness was not significantly associated with PhenoAge acceleration in either the low- or high-GRS group (*β* = −0.029, *t* = −0.97, *p* > 0.05; *β* = −0.017, *t* = −0.66, *p* > 0.05). Overall, the directions of association were similar across GRS strata, particularly for neuroticism, extraversion, and conscientiousness.

## Discussion

This study extends prior research on personality and aging by linking Big Five personality traits to clinical biomarker-based PhenoAge acceleration in a large UK Biobank sample. Biological aging is increasingly understood as a multisystem process that can be captured by biomarkers reflecting physiological dysregulation and disease vulnerability (López-Otín et al., 2013; Kennedy et al., 2014; Jylhävä et al., 2017). PhenoAge and related phenotypic aging measures have been shown to capture morbidity and mortality risks beyond chronological age (Liu et al., 2018; Li et al., 2020). At the same time, personality traits have been linked to health behavior, morbidity, mortality, and aging-related outcomes (Hampson and Friedman, 2008; Roberts et al., 2007; Friedman and Kern, 2014; Stephan et al., 2017). The present findings extend this literature by showing that Big Five personality traits are associated with PhenoAge acceleration and by suggesting that coping-related behavioral regulation partly organizes these associations. The GRS-stratified analyses provided additional context for these associations across different levels of inherited susceptibility to PhenoAge acceleration. Together, these findings suggest that personality traits, coping-related behavioral regulation, and genetic susceptibility may be considered together when examining individual differences in clinical biomarker-based biological aging.

The associations between personality traits and PhenoAge acceleration were not uniform across the Big Five traits. Higher neuroticism scores were associated with higher PhenoAge acceleration. This result is consistent with evidence that neuroticism is linked to poorer physical health, greater stress sensitivity, and less favorable behavioral profiles (Lahey, 2009; Vollrath, 2001; Jokela et al., 2013; Friedman and Kern, 2014). In this study, PhenoAge acceleration captured individual differences in clinical biomarker-based aging beyond chronological age. The positive association with neuroticism may reflect a link between stress-related vulnerability and broader physiological aging profiles. By contrast, higher conscientiousness scores showed one of the strongest inverse associations with PhenoAge acceleration. This pattern is consistent with evidence that conscientiousness is closely related to self-regulation, health-promoting routines, and lower mortality risk (Kern and Friedman, 2008, 2011; Hampson and Friedman, 2008; Turiano et al., 2015). Higher extraversion and agreeableness scores were also associated with lower PhenoAge acceleration, whereas openness was not. Overall, this trait-specific pattern suggests that PhenoAge acceleration may be more closely aligned with personality domains involving emotional reactivity, self-regulation, and interpersonal functioning than with openness (Cacioppo and Cacioppo, 2015; Chapman et al., 2011; Turiano et al., 2012; DeYoung, 2014).

The mediation results help describe the role of coping behaviors in the personality–PhenoAA pattern. Coping behavior partly accounted for the association between neuroticism and higher PhenoAge acceleration. This finding is consistent with stress-process perspectives, which suggest that personality traits are expressed in how individuals appraise, experience, and respond to stressful demands (Lazarus and Folkman, 1984; Bolger and Zuckerman, 1995). Neuroticism has been linked to greater distress, heightened stress sensitivity, and less favorable coping responses in prior research (Connor-Smith and Flachsbart, 2007; Carver and Connor-Smith, 2010). In the present study, higher neuroticism scores were associated with lower coping behavior scores, and this pathway explained a modest portion of the association between neuroticism and PhenoAA. The direct association between neuroticism and PhenoAge acceleration remained after accounting for coping behavior, indicating that additional processes may also be relevant. These processes may include chronic negative affect, repeated stress exposure, sleep disturbance, and physiological stress reactivity (McEwen, 1998; Charles et al., 2013; Wrosch et al., 2008). For extraversion, conscientiousness, and agreeableness, coping behavior partly accounted for their inverse associations with PhenoAge acceleration. The meaning of this mediation pattern differed by trait. Extraversion may be related to more favorable coping profiles through social engagement, positive affect, and greater willingness to seek support. Conscientiousness may be related to PhenoAge acceleration through self-regulation and health-related routines, including avoidance of smoking, maintenance of physical activity, and lower reliance on dysregulated eating or substance-related responses (Kern and Friedman, 2011; Turiano et al., 2015). For agreeableness, the relatively large mediated proportion should be considered alongside its modest total association with PhenoAge acceleration. The coping score represents a composite profile of coping-related behaviors. Overall, these findings suggest that coping-related behavioral profiles may partly account for the associations between personality traits and PhenoAA. Future studies should test pathways for individual components (Folkman and Lazarus, 1980; Carver et al., 1989; Mezuk et al., 2017; Bergmans et al., 2021).

The exploratory GRS-stratified analyses provide additional context for interpreting the personality–PhenoAA associations. The GRS used in this study was constructed from genetic variants previously associated with PhenoAge acceleration and represented inherited susceptibility to this biomarker-based aging phenotype (Kuo et al., 2021). Polygenic and genetic risk scores are increasingly used to examine whether associations between behavioral, psychosocial, or environmental factors differ across levels of inherited susceptibility (Dudbridge, 2013; Watt et al., 2019; Jaramillo-Ospina et al., 2022). Across low- and high-GRS groups, the directions of association were similar for neuroticism, extraversion, and conscientiousness; agreeableness appeared less consistent and openness remained null. This pattern suggests that the main associations between personality traits and PhenoAA were observed across both GRS strata. Taken together, findings from the stratified analyses suggest that these associations can be considered alongside inherited susceptibility. Future studies should test interactions between genetic susceptibility and personality traits more directly.

Several features of the present study strengthen its relevance to research on personality and clinical biomarker-based biological aging. A major strength is the integration of psychological, behavioral, and genetic information within the same analytic framework. Using a large UK Biobank sample, we were able to examine all five Big Five personality traits in relation to PhenoAge acceleration, evaluate coping behavior as a statistical mediator, and further describe these associations across strata of genetic susceptibility (Sudlow et al., 2015). This design allowed us to examine personality–PhenoAA associations together with coping behaviors and genetic susceptibility. It also provided evidence that coping-related behaviors may partly account for these associations and that the observed patterns were broadly similar across inherited risk backgrounds. Another strength is the use of clinical biomarker-based PhenoAge acceleration as the outcome. PhenoAge acceleration reflects a multisystem physiological profile and captures aging-related risk beyond chronological age (Liu et al., 2018; Li et al., 2020). This makes it suitable for examining personality traits, which are broad dispositional characteristics that may relate to aging through multiple behavioral and physiological channels. Multiplicity control and inverse probability weighting sensitivity analyses further supported the robustness of the main mediation patterns, which were broadly similar to the complete-case results.

## Limitations and future research

Several limitations should be noted. First, the cross-sectional design precluded temporal ordering among personality traits, coping behavior, and PhenoAge acceleration. The mediation analyses therefore describe statistical mediation in cross-sectional data. Residual confounding may remain, especially from unmeasured factors such as depressive symptoms, sleep, stress exposure, and other chronic conditions. Second, personality measurement had important limitations. Neuroticism was assessed using a validated short-form measure, whereas extraversion, conscientiousness, agreeableness, and openness were represented by proxy indicators derived from available UK Biobank items. Although these indicators were based on conceptually relevant items and previous research, they have not been extensively validated against established Big Five inventories. Therefore, they may not fully or accurately represent the corresponding traits and may capture only selected aspects of each personality domain. This limitation may have introduced measurement error and affected the estimated associations. Future studies should replicate these findings using validated and comprehensive personality measures. Third, the coping behavior score was based on six behavioral indicators and reflected a composite profile of coping-related behaviors. It did not measure the full range of coping strategies or a single adaptive coping style. Fourth, the primary analyses were based on complete-case data. We compared complete-case participants with those excluded because of missing data and conducted inverse probability weighting sensitivity analyses, which produced results broadly consistent with the main findings. Selection bias related to unmeasured factors may still remain. Fifth, the analysis was restricted to participants of European ancestry to reduce population stratification in the genetic analyses, which may limit generalizability to other populations. Future research could examine more specific psychological and behavioral pathways linking personality to biological aging. Longitudinal studies with repeated measures of personality, coping behavior, and biological aging are needed. Such studies could assess whether coping behavior profiles precede changes in PhenoAge acceleration and whether accelerated biological aging shapes later coping and psychological functioning. Future work could also decompose the coping behavior score into pathways for individual components. Alcohol use, smoking, physical activity, and professional help-seeking may represent different forms of stress regulation and may have different implications for biological aging. Finally, studies that integrate clinical biomarker-based PhenoAge with DNA methylation clocks, immune-cell profiles, and multi-ancestry genetic data could help determine whether personality and coping are related to shared or distinct dimensions of biological aging.

## Conclusion

In this UK Biobank study, higher neuroticism scores were associated with higher clinical biomarker-based PhenoAge acceleration, whereas higher conscientiousness, extraversion, and agreeableness scores were associated with lower PhenoAge acceleration; openness was not clearly associated with PhenoAge acceleration. A coping behavioral profile statistically accounted for a modest part of these associations, and exploratory GRS-stratified analyses suggested broadly similar directions across lower and higher genetic susceptibility groups. This study integrated psychological traits, coping behavior, genetic susceptibility, and PhenoAge acceleration within the same analytic framework. This approach extends personality–aging research beyond single health endpoints and supports a multisystem, biomarker-based view of aging. These findings should be interpreted in light of the cross-sectional design, proxy measures for four traits, a composite coping score, possible residual confounding, complete-case selection, and restricted generalizability beyond European-ancestry participants. Overall, the results support personality-related behavioral regulation as one relevant correlate of biological aging, and longitudinal multi-ancestry studies could clarify temporal ordering and trait-specific pathways.

## Data Availability

Publicly available datasets were analyzed in this study. This research was conducted using the UK Biobank Resource under Application Number 44430. The UK Biobank data are available on application to the UK Biobank (www.ukbiobank.ac.uk/).
