# Supplementary material for: Personality traits and biological aging: the mediating role of coping behavior
Source: Front Psychol. 2026 Oct 7;17:1876764. doi: 10.3389/fpsyg.2026.1876764 (PMC13683731; doi:10.3389/fpsyg.2026.1876764)
Supplement: Supplementary file 1 [file Table_2.DOCX]

Supplementary Table S2. Complete cross-sectional statistical mediation results

| **Trait** | **Effect** | **Beta** | **SE** | **P value** | **95% CI** |
| --- | --- | --- | --- | --- | --- |
| Neuroticism | a path: trait -> coping | -0.010 | 0.002 | 8.45e-06 | [-0.014, -0.006] |
| Neuroticism | b path: coping -> residualized PhenoAA | -0.577 | 0.015 | 0 | [-0.605, -0.548] |
| Neuroticism | Direct association | 0.032 | 0.011 | 0.004807 | [0.010, 0.054] |
| Neuroticism | Indirect association | 0.006 | 0.001 | 9.615e-06 | [0.003, 0.008] |
| Neuroticism | Total association | 0.038 | 0.011 | 0.0009331 | [0.015, 0.060] |
| Neuroticism | Proportion statistically accounted for | 15.4% | 0.055 | 0.004988 | [0.046, 0.261] |
| Sociability / Extraversion | a path: trait -> coping | 0.105 | 0.003 | 0 | [0.099, 0.112] |
| Sociability / Extraversion | b path: coping -> residualized PhenoAA | -0.556 | 0.015 | 0 | [-0.585, -0.528] |
| Sociability / Extraversion | Direct association | -0.243 | 0.016 | 0 | [-0.274, -0.211] |
| Sociability / Extraversion | Indirect association | -0.059 | 0.002 | 0 | [-0.063, -0.054] |
| Sociability / Extraversion | Total association | -0.301 | 0.016 | 0 | [-0.333, -0.270] |
| Sociability / Extraversion | Proportion statistically accounted for | 19.5% | 0.012 | 0 | [0.170, 0.219] |
| Diligence / Conscientiousness | a path: trait -> coping | 0.069 | 0.003 | 0 | [0.063, 0.075] |
| Diligence / Conscientiousness | b path: coping -> residualized PhenoAA | -0.562 | 0.015 | 0 | [-0.590, -0.533] |
| Diligence / Conscientiousness | Direct association | -0.275 | 0.016 | 0 | [-0.307, -0.244] |
| Diligence / Conscientiousness | Indirect association | -0.039 | 0.002 | 0 | [-0.043, -0.035] |
| Diligence / Conscientiousness | Total association | -0.314 | 0.016 | 0 | [-0.345, -0.283] |
| Diligence / Conscientiousness | Proportion statistically accounted for | 12.3% | 0.009 | 0 | [0.106, 0.140] |
| Warmth / Agreeableness | a path: trait -> coping | 0.018 | 0.002 | 6.153e-16 | [0.014, 0.022] |
| Warmth / Agreeableness | b path: coping -> residualized PhenoAA | -0.576 | 0.015 | 0 | [-0.605, -0.548] |
| Warmth / Agreeableness | Direct association | -0.026 | 0.011 | 0.01803 | [-0.048, -0.005] |
| Warmth / Agreeableness | Indirect association | -0.010 | 0.001 | 2.314e-15 | [-0.013, -0.008] |
| Warmth / Agreeableness | Total association | -0.037 | 0.011 | 0.001065 | [-0.059, -0.015] |
| Warmth / Agreeableness | Proportion statistically accounted for | 28.2% | 0.089 | 0.001623 | [0.107, 0.457] |
| Curiosity / Openness | a path: trait -> coping | -0.003 | 0.004 | 0.4442 | [-0.011, 0.005] |
| Curiosity / Openness | b path: coping -> residualized PhenoAA | -0.577 | 0.015 | 0 | [-0.606, -0.549] |
| Curiosity / Openness | Direct association | -0.024 | 0.020 | 0.2161 | [-0.063, 0.014] |
| Curiosity / Openness | Indirect association | 0.002 | 0.002 | 0.4443 | [-0.003, 0.006] |
| Curiosity / Openness | Total association | -0.023 | 0.020 | 0.2537 | [-0.061, 0.016] |
| Curiosity / Openness | Proportion statistically accounted for | -7.7% | 0.127 | 0.5456 | [-0.325, 0.172] |

Note. The outcome was PhenoAge acceleration (PhenoAA), calculated as the residual of PhenoAge regressed on chronological age. Models were adjusted for age, sex, Townsend deprivation index, coronary heart disease, hypertension, and diabetes. The a path represents the association between each personality trait and coping behavior; the b path represents the association between coping behavior and PhenoAge acceleration adjusted for the personality trait and covariates.
